# Classification of breast cancer patients using somatic mutation profiles and machine learning approaches

**DOI:** 10.1186/s12918-016-0306-z

**Published:** 2016-08-26

**Authors:** Suleyman Vural, Xiaosheng Wang, Chittibabu Guda

**Affiliations:** 1 Department of Genetics, Cell Biology and Anatomy, University of Nebraska Medical Center, Omaha, NE 68198 USA; 2 School of Basic Medicine and Clinic Pharmacy, China Pharmaceutical University, Nanjing, 211198 China; 3 Bioinformatics and Systems Biology Core, University of Nebraska Medical Center, Omaha, NE 68198 USA; 4 Department of Biochemistry and Molecular Biology, University of Nebraska Medical Center, Omaha, NE 68198 USA; 5 Fred and Pamela Buffet Cancer Center, University of Nebraska Medical Center, Omaha, NE 68198 USA

**Keywords:** Unsupervised and supervised machine learning, Gene mutation profiles, TCGA, Breast cancer classification, Breast cancer subtypes, Cancer stage prediction, Whole exome sequencing data analysis

## Abstract

**Background:**

The high degree of heterogeneity observed in breast cancers makes it very difficult to classify the cancer patients into distinct clinical subgroups and consequently limits the ability to devise effective therapeutic strategies. Several classification strategies based on ER/PR/HER2 expression or the expression profiles of a panel of genes have helped, but such methods often produce misleading results due to their dynamic nature. In contrast, somatic DNA mutations are relatively stable and lead to initiation and progression of many sporadic cancers. Hence in this study, we explore the use of gene mutation profiles to classify, characterize and predict the subgroups of breast cancers.

**Results:**

We analyzed the whole exome sequencing data from 358 ethnically similar breast cancer patients in The Cancer Genome Atlas (TCGA) project. Somatic and non-synonymous single nucleotide variants identified from each patient were assigned a quantitative score (C-score) that represents the extent of negative impact on the gene function. Using these scores with non-negative matrix factorization method, we clustered the patients into three subgroups. By comparing the clinical stage of patients, we identified an early-stage-enriched and a late-stage-enriched subgroup. Comparison of the mutation scores of early and late-stage-enriched subgroups identified 358 genes that carry significantly higher mutations rates in the late stage subgroup. Functional characterization of these genes revealed important functional gene families that carry a heavy mutational load in the late state rich subgroup of patients. Finally, using the identified subgroups, we also developed a supervised classification model to predict the stage of the patients.

**Conclusions:**

This study demonstrates that gene mutation profiles can be effectively used with unsupervised machine-learning methods to identify clinically distinguishable breast cancer subgroups. The classification model developed in this method could provide a reasonable prediction of the cancer patients’ stage solely based on their mutation profiles. This study represents the first use of only somatic mutation profile data to identify and predict breast cancer subgroups and this generic methodology can also be applied to other cancer datasets.

**Electronic supplementary material:**

The online version of this article (doi:10.1186/s12918-016-0306-z) contains supplementary material, which is available to authorized users.

## Background

Breast cancer (BC) is a genetically and clinically heterogeneous disease; hence, the effectiveness of a specific treatment greatly varies among BC patients. There have been several widely accepted methods to classify breast cancers into distinct subtypes [1–7], such as histopathological classification based on the morphological features, and analysis of the presence or absence of immunohistochemical (IHC) markers like ER, PR and HER2. In addition, application of unbiased hierarchical clustering on gene expression assays has led to the identification of five distinct breast cancer mRNA subtypes: luminal A, luminal B, HER2 overexpression, basal-like and normal breast tissue-like [2]. The differences in gene expression patterns in these subtypes reflect the basic alterations in the cell biology of the tumor and are associated with significant variation in clinical outcome such as overall survival and disease free survival [8]. Particularly, Luminal A subtype patients are found to have relatively better prognosis while basal-like subtype patients having the worst prognosis. Importantly, this molecular classification has successfully discovered sub-classes of ER-positive and/or PR-positive breast cancers as luminal A and luminal B. This is a significant achievement because even though clinical assessment of IHC utilizes ER, PR, and HER2 status, these markers could not let the separation of these two distinct subtypes which have very different clinical outcomes [3, 8].

Currently, the microarray-based BC classification has been regarded as the gold standard [9]. However, the main limitation of this method is its inability to assign samples consistently to specific molecular subtypes [10–12]. A main reason is that the dynamic nature of gene expression within an individual may yield misleading results for classification. In contrast, gene mutations at DNA level can be stably detected. As all cancers carry somatic mutations in their genomes and mutational heterogeneity widely exists in cancers, classification of cancers based on the mutation profile could be useful for cancer diagnosis and treatment. On the other hand, with the advancement of new sequencing technologies, genome sequencing has become affordable for routine diagnostic purposes. Hence, exploration of cancer classification based on gene mutation profiles and incorporation of the classification into the clinical decision support system could be meaningful for personalized care of cancer patients.

Several studies that integrated multiple types of molecular data for breast cancer clustering have been proposed. Curtis et al. [13] suggested a novel molecular stratification of breast cancer by combining genome and transcriptome assessments of 2000 breast cancer patients. Based on the impact of somatic copy number aberrations on the transcriptome, they revealed novel subgroups of breast cancers. Likewise, Ali et al. [5] classified breast cancers into ten subtypes based on the integration of genomic (copy number variation) and transcriptomic (gene expression) data. And in another study [6], the authors proposed a computational method that combined gene expression and DNA methylation data to implement machine learning aided classification of breast cancer patients. In a more recent study [7], the authors proposed a network-based stratification method to classify cancers by combining somatic mutation profiles with gene interaction networks, and identified four subtypes of breast cancers.

It is often difficult to predict the impact of single nucleotide mutations in the genome at a molecular level and consequently their effect on cancer initiation and progression. In addition, somatic mutations are often sparsely distributed in different cancer samples. Therefore, previous studies used somatic mutation data as an auxiliary information in combination with other data types to classify cancer and/or used as a binary entity (the presence or absence of a mutation) [7]. This strategy is over simplified, given the fact that all mutations are not identical and their impact on the clinical outcome often broadly varies based on many factors such as the genomic location of mutations (coding vs. non-coding), perturbing the mRNA transcription (stop-gain or stop-loss mutations, frame shifts, etc.) or altering the amino acids (synonymous vs. non-synonymous) in the encoded proteins. Hence, quantification of the deleterious impact of mutations on the gene function, and the use of this information in the mutation-based clustering scheme could yield meaningful results.

In this study, we developed a novel method to classify breast cancers based on the quantification of somatic mutation profiles. We analyzed the whole exome sequencing data from 358 ethnically similar BC patients in The Cancer Genome Atlas (TCGA) project. We first scored the functional impact of each variant using Combined Annotation–Dependent Depletion (CADD) scores [14], and then clustered the 358 BC patients into three subgroups using the Non-negative Matrix Factorization (NMF) method. Furthermore, we investigated the biological implications of the classes that we discovered in this study. Finally, we developed a computational model to predict the subgroup of the BC patients using supervised machine learning methods. The approach presented in this study exhibits a generic methodology that might be applied for classification of other cancer types.

## Results and discussion

### Data representation and challenges

**Fig. 1:**
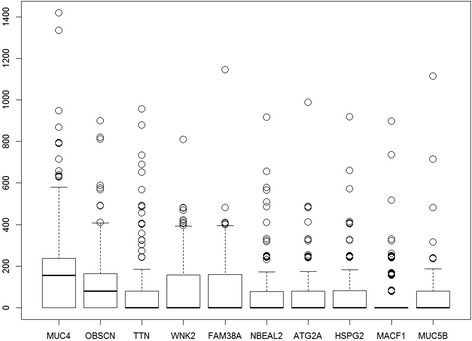
Distribution of total mutational scores for the top ten variant genes. The top 10 most heavily mutated genes include several proven cancer associated genes including *MUC4* and *OBSCN*

Our initial observation on the mutation score matrix showed that, the C-scores range from 0 to 1417.14 and distribution of scores for top ten variant genes can be seen in Fig. 1. Comparison against the COSMIC database shows that nine out of these ten genes (with the exception of FAM38A gene) have evidence of abundant accumulation of somatic mutations in large population screens [15].

Somatic mutation profiles of BC patients exhibit a very sparse data form, unlike other data types such as gene expression or methylation in which nearly all genes or markers are assigned a quantitative value in all the patients. Even clinically identical patients may share no more than a single mutation [16–18]. Therefore, this problem introduces too many zero valued entries to the main data structure (96 %). On the other hand, from machine learning perspective, having a limited number of patients (a far less number of patients than the number of effected genes in a cohort) introduces a dimensionality challenge commonly known as the “curse of dimensionality” in machine learning. In this study, we are faced with this challenge as we observed the sample-to-feature ratio of 1:50 (358/18117) in the main data structure.

In order to overcome the aforementioned challenges, generally there are two popular approaches, namely; feature extraction and feature selection. Feature extraction transforms the current existing features into a lower dimensional space and widely used example methods include principal component analysis (PCA) and linear discriminant analysis (LDA), while feature selection selects a subset of features without applying any transformation. These methods increase the sample-to-feature ratio and decrease the sparseness hence making the clustering both feasible and more effective. In this study, we used feature selection by ranking the features (genes) in decreasing order of their variance value and selected top *n* features for clustering (see methods for more details). We optimized the size of n to be 854 genes in our clustering method.

### Classification of breast cancers based on somatic mutations

Unsupervised clustering is the task of grouping a set of samples that have no label information, which results in grouping samples in such a way that samples in the same group are more similar in a specified measure to each other than to those in the other groups. There are several methods trying to achieve this goal such as k-means clustering, hierarchical clustering and expectation maximization (EM) algorithms. However, these methods perform poorly or cannot come to a solution when applied to sparse data, as is the case in our study. Therefore, we selected to use NMF because of its proven superior performance when tested on biological data based applications [19–21]. NMF was introduced in its modern formulation by Lee and Seung [21] as a method to decompose images.

As a factorization method, NMF algorithm takes our mutation score matrix as the input and decomposes it to two smaller matrices (basis matrix W and coefficient matrix H). The output coefficient matrix (matrix H) is used to make sample cluster assignments. Refer to methods for more details.

Using the NMF clustering algorithm on our dataset, we stably clustered the samples into three groups using the top 854 genes, which have the highest variance values of mutation scores across all the samples. The three groups Cluster 1, 2, and 3 involve 169, 121 and 68 patients, respectively. Refer to methods section for more details.

**Fig. 2:**
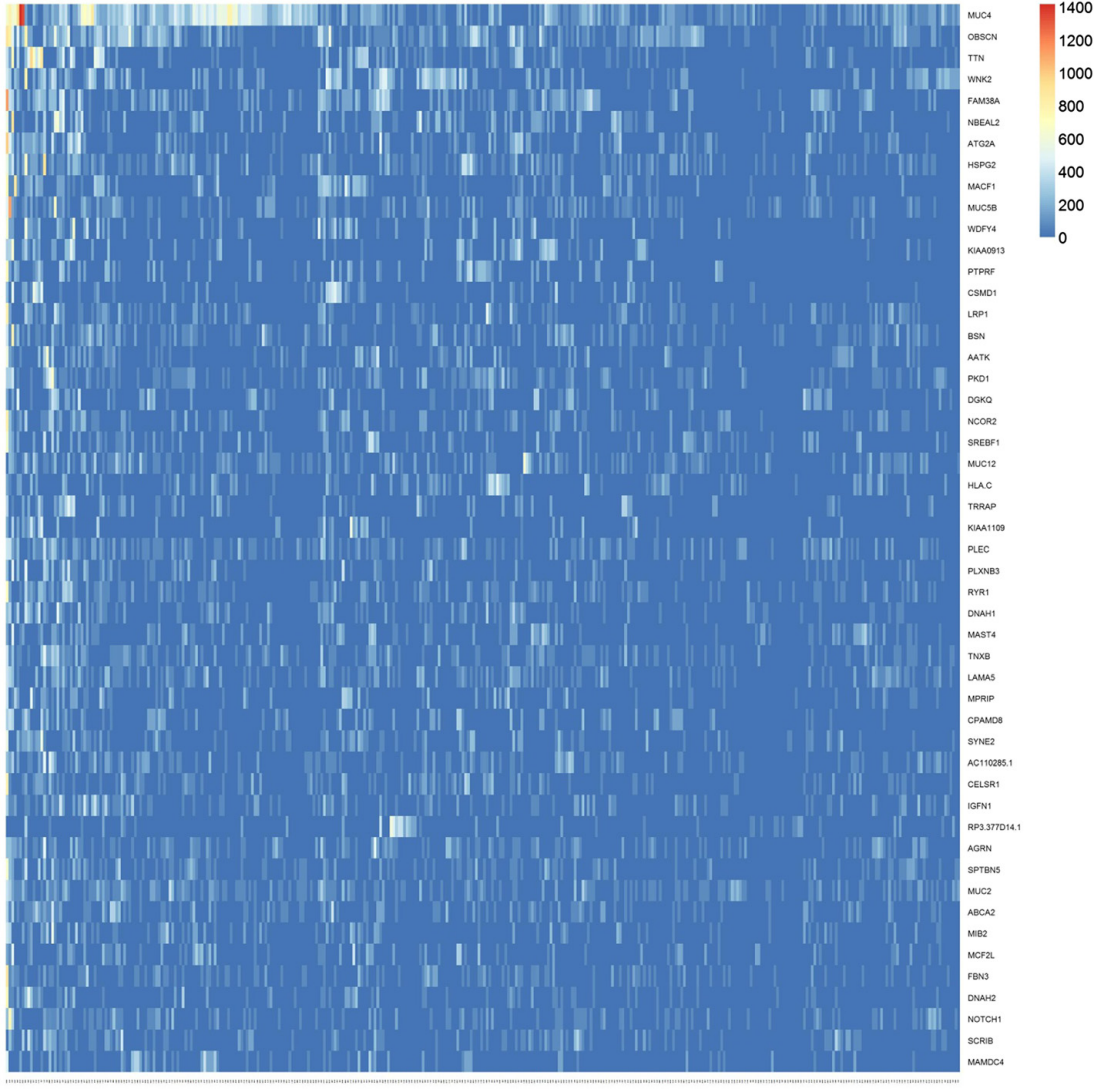
Input matrix with C-scores of the top 50 mutated genes. The heat map shows the most heavily mutated 50 genes. The columns represent patients (358) and rows represent genes. One of the challenge of the dataset is being extremely sparse which can be seen in the heat map as most of the cells are colored very close to blue, which indicates a 0 (C-score) mutation score, with the exception of the first few columns. We identified that the main data structure is composed of 96 % zeros

**Fig. 3:**
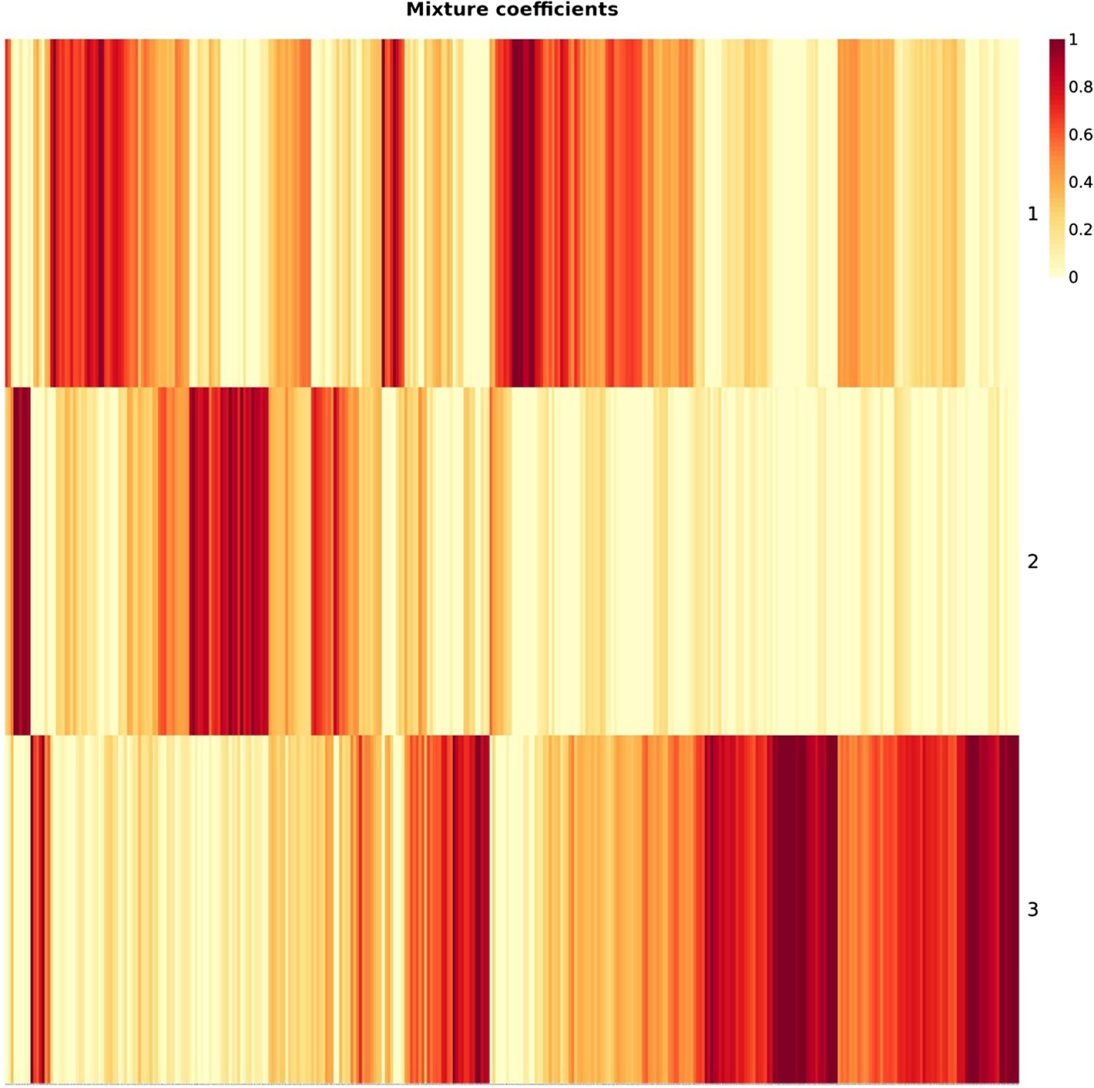
Coefficient matrix (H). The coefficient matrix (H), 3 × 358 in size, is used for assigning samples to clusters. The columns of the matrix represent patients and rows represent metagenes. We generated 3 metagenes that are used to cluster patients into 3 groups. The number of metagenes (rank of clustering) is determined by running the algorithm iteratively over a range of biologically reasonable parameters as explained in methods section

In Fig. 2, we show a representation of the input data in the mutation score matrix, focusing only the top 50 variant genes for illustration purpose. As it can be seen, data represents a very sparse form (most of the cells are colored blue meaning a zero score) which makes most clustering approaches inapplicable. Additional file 1: Figure S1 and Fig. 3 are the output matrices from decomposition of the mutation score matrix, which we input to NMF algorithm. Note that multiplication of the two output matrices will approximately yield the input data. In Additional file 1: Figure S1, we see the basis matrix (W), which is not used in the scope of this study; however it could serve for clustering purpose of the genes. Figure 3 displays the coefficient matrix (H), where the rows represent the metagenes that are a compact representation of all the genes, and columns represent the patients. We use this matrix to make sample to cluster associations by assigning the samples to the clusters where we observe the highest metagene value, i.e., the dark red color, (See methods section for details).

**Fig. 4:**
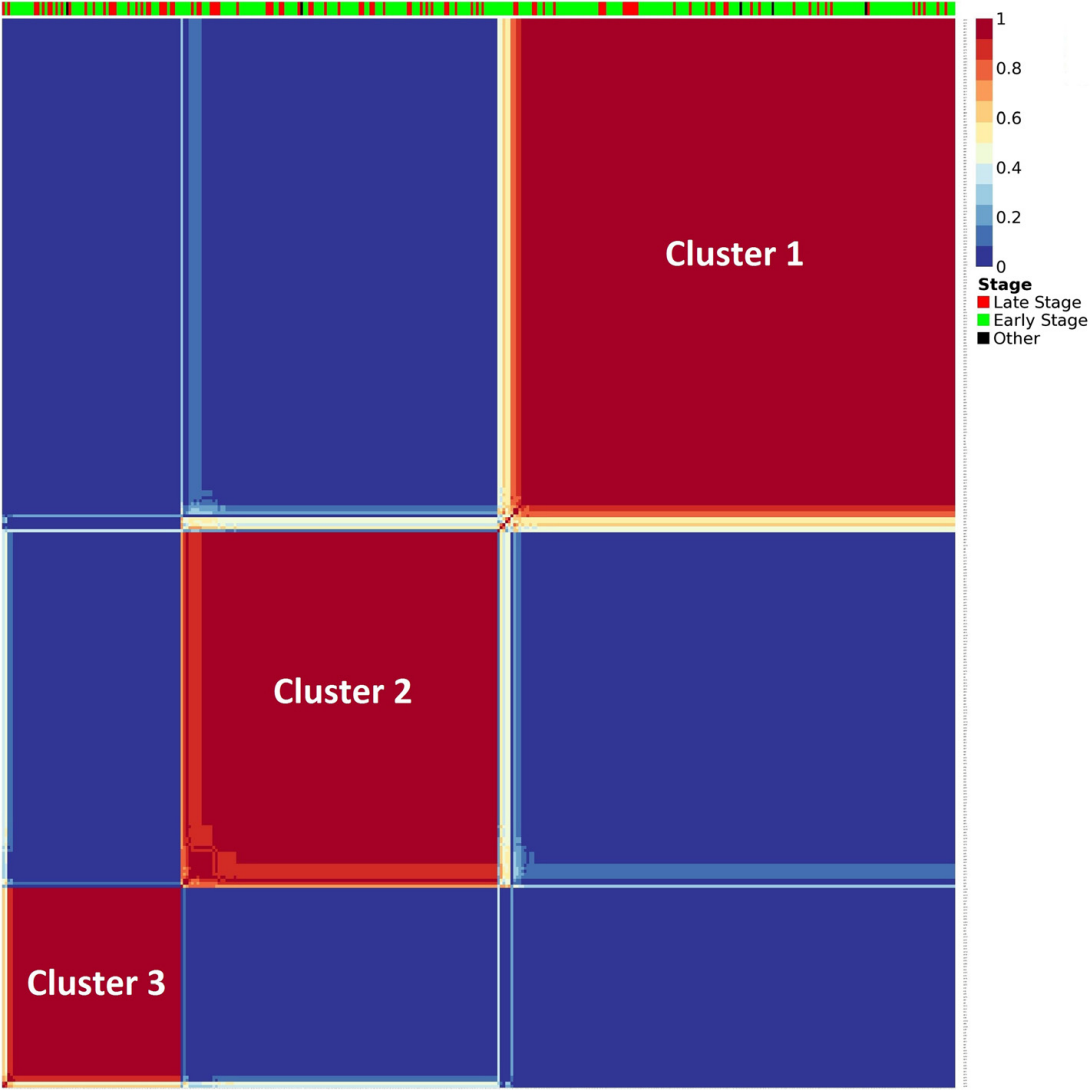
Consensus matrix. The consensus matrix is 358 × 358 in size and illustrating the stability of the clustering. In ideal case, all the entries are expected to be either 0 or 1, making solid colored blocks. The bar on top indicates the clinical stage of each patient. The Silhouette score of this matrix is 0.958 which indicates a very stable clustering. (Silhouette (consensus) = 0.958)

Figure 4 illustrates the stability of the clustering by displaying the consensus matrix, which was generated after 100 NMF runs using Brunet’s [22] approach (explained in methods section). We used the silhouette score of consensus matrix to determine the optimum number of genes and clusters. In an ideal clustering case, we expect to observe values either close to 1 or 0, indicating the probability of two samples being in the same cluster or not, respectively, which displays solid colored blocks. A value of one represents the highest probability that two samples are in the same cluster (red blocks) and the value of zero denotes the opposite (blue blocks). In Fig. 4 it can be seen that the dataset is clearly clustered into three distinct groups.

### Characterization of discovered clusters

We investigate the clinical significance of discovered clusters by comparing the BC stage of the patients in each cluster. For this purpose, we analyze the distribution of patients according to their disease stage provided in the TCGA data. We found that Cluster 1 was dominated by early stage patients while Cluster 3 had much higher proportion of late stage patients compared to Cluster 1 (Fisher’s exact test p-value = 0.02048, Table 1). As can be seen in Table 1, the number distribution of patients in each cluster with stage ratio (number of early stage patients over late stage patients) for Cluster1 is more than two-fold higher than that of Cluster 3; hence here we call Cluster 1 as the early-stage-enriched cluster, Cluster 2 as the mixed cluster and Cluster 3 as the late-stage-enriched cluster. This separation of patients by their disease stage indicates that our clustering method can successfully discriminate breast cancer patients by their disease stage using only the somatic mutational profiles of patients from their exome sequencing data.

**Table 1:** Distribution of patients in the clusters discovered

Cluster	Number of patients^a^	Number of early stage patients^b^	Number of late stage patients^c^	Ratio^d^
Cluster 1	166	131	35	3.74
Cluster 2	120	86	34	2.53
Cluster 3	67	41	26	1.58

^a^Five patients were not included due to their unknown stage information

^b^Sum of stage I and II patients in each cluster

^c^Sum of stage III and IV patients in each cluster

^d^Ratio of the number of early stage patients to the number of late stage patients

Next, we compared the somatic mutation profiles of patients between the early and late-stage-enriched clusters (Cluster 1 vs. Cluster 3). We found that there were 358 genes, which have significantly higher mean mutation scores in the late-stage-enriched cluster (Cluster 3) than in the early-stage-enriched cluster (Cluster 1) (Wilcox rank-sum test, FDR < 0.1), but none of the genes have significantly higher mean mutation scores in Cluster 1 than in Cluster 3. This interesting finding indicates that these genes may have accumulated deleterious mutations leading to the progression of breast cancer into advanced disease states. We identified that tumor suppressor genes, APC, BRCA2; and oncogene, MLL are among the 358 genes used in this comparison. Table 2 shows the top 25 most significant genes that are found to show significantly higher mutation rates in late-stage-enriched cluster.

**Table 2:** Most significant 25 genes that show higher mutation rates in late-stage-enriched cluster (cluster 3)

Gene symbol	*p* value	FDR value
TTN	0	0
MACF1	0	0
FSIP2	0	0
DNAH9	0	0
DST	0	0
KIAA1731	0	0
DSP	0	0
VPS13D	4.44E-16	4.74E-14
UBR4	1.55E-15	1.47E-13
C10ORF18	1.89E-15	1.61E-13
SYNE1	2.55E-15	1.98E-13
HERC1	5.44E-15	3.87E-13
CSMD1	2.35E-14	1.55E-12
CHD9	2.93E-14	1.79E-12
KIAA1109	3.26E-14	1.86E-12
XIRP2	4.04E-14	2.16E-12
APC	8.06E-14	4.05E-12
GPR98	1.23E-13	5.86E-12
DOCK9	4.62E-13	2.07E-11
VCAN	5.78E-13	2.47E-11
SYNE2	7.90E-13	3.21E-11
RIF1	1.06E-12	4.10E-11
NOTCH2	1.40E-12	5.21E-11
WDFY4	1.70E-12	6.05E-11
MLL	2.28E-12	7.79E-11

We stratified the 358 genes into different gene families using the Gene Set Enrichment Analysis (GSEA) [23] tool as shown in Table 3. We observe that a significant proportion of the genes belong to transcription factor and protein kinase gene families, which are well known to be related to the progression of BC [24, 25]. Table 4 shows the assignment of these genes to functionally distinct gene families.

**Table 3:** GSEA classification of 358 genes that have significantly higher mean mutation scores in cluster 3 compared to cluster 1

GSEA gene families	Cytokines/growth factors	Transcription factors	Homeodomain proteins	Cell differentiation markers	Protein kinases	Translocated cancer genes	Oncogenes	Tumor suppressors
Tumor suppressors	0	1	0	0	0	1	0	4
Oncogenes	0	3	0	0	0	11	12	
Translocated cancer genes	0	4	0	0	0	12		
Protein kinases	0	0	0	1	16			
Cell differentiation markers	0	0	0	4				
Homeodomain proteins	0	3	3					
Transcription factors	0	25						
Cytokines and growth factors	3							

Note that some of the genes in our gene list are not found in any GSEA (Gene Set Enrichment Analysis) gene family

**Table 4:** Distribution of genes to functionally distinct gene families, by GSEA

Transcription factors	Protein kinases	Translocated cancer genes	Oncogenes	Cell differentiation markers	Tumor suppressors	Homeodomain proteins	Cytokines and growth factors
ARID1B	ALPK3	AKAP9	AKAP9	CD44	APC	CUX2	LTBP3
BPTF	CDK20	CASC5	CASC5	ITGA2B	BRCA2	ZFHX3	SEMA5B
BRD1	CIT	EP300	MLL	L1CAM	EP300	ZFHX4	TG
BRPF1	EPHA1	MLL	MLLT4	MST1R	FANCA		
CASZ1	GUCY2D	MLLT4	MYH9				
CHD3	IRAK1	MYH9	NACA				
CUX2	KALRN	NACA	NOTCH2				
EP300	LRRK1	NUMA1	NUMA1				
HIVEP1	MST1R	NUP98	NUP98				
LMO7	PRKDC	RNF213	RNF213				
MED12	RPS6KA4	TET1	TET1				
MGA	SPEG	WHSC1	WHSC1				
MLL	STK36						
MLLT4	TRRAP						
NCOR2	TTN						
PHF3	WNK1						
RERE							
SALL2							
SF1							
SPEN							
SREBF2							
UBR4							
WHSC1							
ZFHX3							
ZFHX4							

### Network analysis of differentially mutated genes

**Fig. 5:**
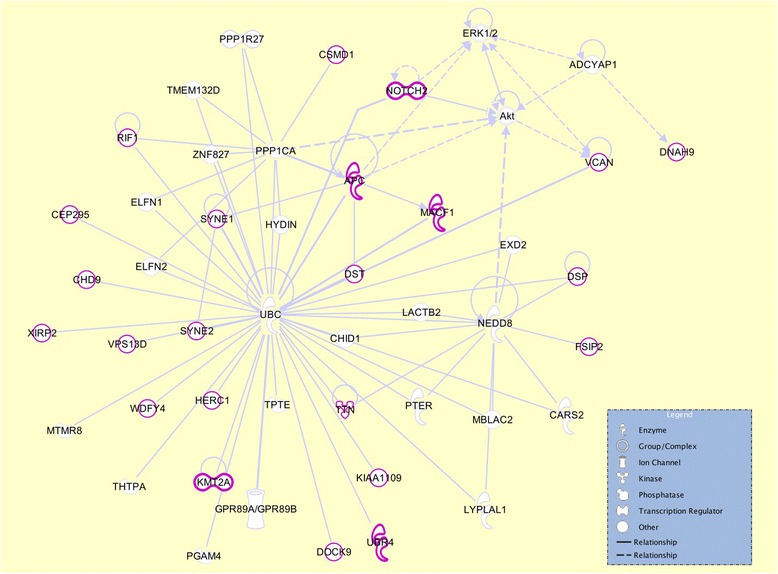
Interaction network analysis of the top 25 genes. The image shows the interactions of the top 25 genes with highest mutation load in the late-stage-enriched cluster compared to the early-stage-enriched cluster of patients

We carried out the network analysis of the top 25 highly mutated genes (Table 2) in the late-stage-enriched cluster compared to the early-stage-enriched cluster patients, to understand the functional relationship among these genes. The network in Fig. 5, generated using the Ingenuity Pathway Analysis (IPA) program shows several interaction hubs, where the genes highlighted in purple color are highly mutated in the late stage cluster patients. Most of the genes in our list interact with the central hub protein, UBC, which is expected because most of the proteins (especially the unneeded or damaged ones) are ubiquitinated before proteosomal degradation. It has been known that ubiquitin-proteasome system regulates the degradation of a number of cancer-associated genes [24]. APC (adenomatous polyposis coli) is another key tumor suppressor seen in this network that acts as an antagonist of the Wnt signaling pathway, with a number of roles in cancer development and progression such as cell migration, adhesion, apoptosis, etc. The role of APC mutations in breast cancers has been well documented in the literature [25]. It is noteworthy to mention two transcriptional regulator genes in our list, NOTCH2 and KMT2A (MLL). NOTCH2 is a key regulator of Akt, and its role is well documented in several cancers including in apoptosis, proliferation and epithelial-mesenchymal transition (EMT) pathway [26]. Several somatic mutations in NOTCH2 are also associated with different cancers in COSMIC database [27]. MLL is a transcriptional regulator and an oncogene with a variety of roles in cell proliferation and apoptosis [28].

### Class prediction of breast cancers based on somatic mutations

Using the aforementioned BC clusters, we labeled each sample with its assigned cluster, and developed a classification model to see how accurate we can predict clusters of unseen breast cancer patients based on their somatic mutations. With this model, we can predict the cluster of an unseen patient, using his/her mutation profile; hence we get insight about the patient’s clinical outcome, like BC stage. As an example; if the model predicts a new patient to be in the Cluster3, than we can expect this patient to be in late stage with certain genes be more likely to carry higher mutation loads.

We labeled each patient with its assigned cluster and tested five popular machine learning (ML) algorithms; Random Forest (RF) [15], Support Vector Machine (SVM) [29], C4.5 [30], Naïve Bayes [31], and k-Nearest Neighbor(KNN) [32] to find the most appropriate algorithm for our dataset.

We used a 10-fold cross-validation for evaluation of classifier performances. In each loop of the 10-fold cross validation, after withdrawal of the test set, we did feature selection using the information gain feature selection method [33] and selected the top 500 genes, which provide the highest information gain based on the training set. Therefore, in total, we selected ten sets of 500 genes in the 10-fold cross validation. Out of the aforementioned ML algorithms, we selected to further use the RF method in this study as it achieved the best 10-fold cross-validation accuracy with 70.86 %. We believe that the sparseness of the data along with the low sample to feature ratio and difficulty of multiclass prediction are the reasons behind this moderate accuracy.

Also we observe that SVM algorithms achieved a very close accuracy but with a loss in TPR, FPR and F measure. And KNN method yielded the worst accuracy of all the methods we used. Table 5 shows the performance measures of each ML algorithm.

**Table 5:** 10-fold cross-validation performance results of five classifiers

Classifier	Accuracy	TPR	FPR	TNR	FNR	F measure
Random forest	70.86	0.58	0.19	0.81	0.42	0.59
Support vector machine	69.16	0.49	0.16	0.84	0.51	0.53
J48 (C4.5)	60.11	0.47	0.26	0.74	0.53	0.47
Naïve Bayes	57.24	0.45	0.29	0.71	0.55	0.44
k-Nearest Neighbors	49.17	0.25	0.16	0.84	0.75	0.31

**Fig. 6:**
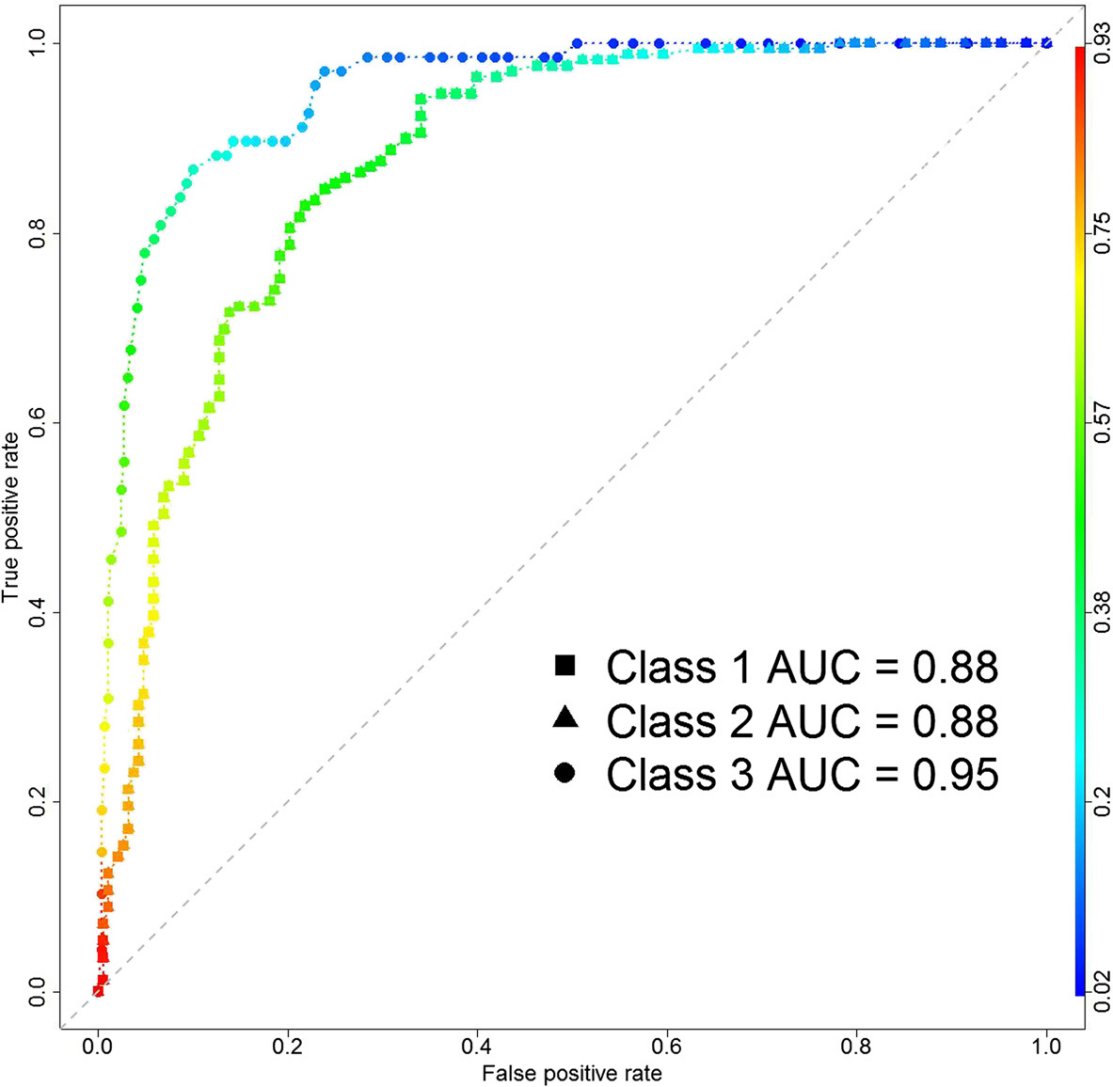
ROC curves. The ROC curve, which is used to show the accuracy of the predictions made by the model, shows the relationship between TPR (sensitivity) and FPR (1-specificity) for each class. As the AUC values indicate that the prediction model achieves a better accuracy in discrimination of early-stage-enriched and late-stage-enriched classes with 0.88 and 0.95 respectively

Figure 6 shows the receiver operating characteristic (ROC) curves for each class that illustrate the relationship between TPR (sensitivity) and FPR (1-specificity) for each class. In the perfect case, an ROC curve goes straight up on the Y-axis and then to the right parallel to the X-axis; thus maximizing the area under the curve (AUC). An AUC close to one indicates that the classifier is predicting with maximum TP and minimum FP. We calculated the AUC for clusters 1, 2 and 3 (used interchangeably as class in this section) as 0.88, 0.8 and 0.95, respectively, indicating that the classification model can better differentiate the late stage patients against the remaining patients.

We also used a permutation test, by running the same class prediction procedure with RF on 10,000 randomly labeled datasets and none of the 10-fold cross-validations gave us a better accuracy, yielding a very significant p-value (*p*-value < 10^−4^) (see methods for more details). This supports the robustness of our model and the predication accuracy.

## Conclusions

Breast cancers are highly heterogeneous diseases; therefore, accurate classification of BCs is an important step towards making accurate treatment decisions. Next generation sequencing opens new venues to better understand the genomic background of BC. In this study, we developed a novel BC classification system that solely uses somatic mutational profiles of BC patients, generated by whole exome sequencing, to identify clinically differentiable subgroups together with a class prediction model.

We used the TCGA breast cancer somatic mutation dataset including 358 patients and applied necessary filtration to the reported variations. Following, we used NMF clustering method to discover subgroups in the dataset, which yielded 3 clustered groups of patients. We investigated the clinical significance of discovered clusters by comparing the BC stage of the patients in the clusters and found that there exists a significant separation of patients according to their disease stage; hence we named Cluster 1 as early-stage-enriched and Cluster 3 as late stage rich. Then we compared the mean mutation scores of early and late-stage-enriched clusters and found that late-stage-enriched cluster patients carry a significantly higher rate of mutations in 358 genes. We also identified important networks, biological functions and pathways regulated by these genes. Finally, we used RF classification algorithms to develop a classification model, to make cluster predictions for unknown BC patients hence can provide insights about the disease stage and significantly mutated genes.

In conclusion, this study demonstrates that clinically distinguishable breast cancer subtypes can be identified solely based on somatic mutation profile data from breast cancer patients. Further, our classification model can be used to predict the unknown subtypes of breast cancers, given the somatic mutation profile of a patient. This generic methodology can also be applied to classify and predict other cancer types.

## Methods

### Datasets, representation and reference databases

We downloaded the sequence variation data in variant call format (vcf) for the TCGA breast cancer whole exome sequencing data. To eliminate the population heterogeneity effect, we selected the breast cancer patients (*n* = 358) from white, not Hispanic or Latino group for analysis. We obtained an average of 17,640 point variations per patient, generated by VarScan2 [34], a highly sensitive tool to detection of somatic mutations in exome sequencing data from normal-tumor pairs.

In this study we used CADD, a method that integrates functional annotations, conservation, and gene-model information into a single score called C-score. As mentioned in the original publication, [14] C-scores correlate with allelic diversity, annotations of functionality, pathogenicity, disease severity, experimentally measured regulatory effects, and complex trait associations. This score is originally defined to range from negative infinity to positive infinity, where higher score denotes more deleterious effects; however since our clustering (NMF) algorithm requires all data entries to be positive, we transformed all the scores by adding the minimum score to the original scores.

In addition we used dbSNP data [35] to exclude commonly-found population polymorphisms. Lastly, we used Database of Human Non-synonymous SNVs and Their Functional Predictions and Annotations (dbNSFP) [36] to retrieve CADD scores of mutations.

Our method uses an extensive data structure (mutation score matrix) to keep track of all the deleteriousness scores (C-scores) of somatic mutations used for machine learning. The mutation score matrix represents a table that contains the genes in rows and the patients in columns, yielding a matrix of size 18,117 rows by 358 columns, with at least one mutation in each row. And each cell contains the sum of all C-scores of mutations found in a gene for a patient.

### Exome data analysis and variant calling

We have obtained an average of 17,640 point variations per patient generated by VarScan2 [34] and applied a set of filters to select only those that are likely to exhibit an impact on the function and/or the structure of the gene or protein. Since the generation of next-generation sequencing (NGS) data and variant calling involves several error prone steps, filtration of the variant data constitutes a major step in variant analysis. Firstly, we focus only on the somatic (non-inherited) and nonsynonymous (causing a change in the translated amino acid) point mutations because of their perceived impact on disease initiation and progression. Secondly, even though exome sequencing targets only the coding regions of DNA, the exome capture kits often amplify off-target non-coding regions such as intergenic, untranslated and intron regions. Hence, we filter out all the variations outside of the coding region. We analyze the remaining variations by their impact on the function or structure of the resulting protein. Finally, we check the population frequency of remaining variations in Single Nucleotide Polymorphism Database (dbSNP) [35], which is a public achieve for genetic variation developed and hosted by National Center for Biotechnology Information (NCBI). In this step, we filter out the variations that are commonly found in population and hence are not necessarily associated with a disease. Generally, variations with less than 0.05 minor allele frequency (MAF) are considered as phenotype-causing variations and hence are called as mutations.

### Clustering

We implemented an *m* × *n* mutation score matrix to keep track of the sum of the variant scores in all genes, where *m* is the number of genes (18,117) and *n* is the number of samples (358 patients). The value in entry (*i*, *j*) indicates the mutation score of gene *i* in sample *j*, which is the sum of all C-scores of mutations found in the gene *i* for the sample *j*.

Due to the number of features (tens of thousands genes) being much more than the number of samples (hundreds of samples), we first used feature selection to select only the informative features for clustering; thus to reduce the feature size. We ranked the features in decreasing order of their variance values (Equation 1) and selected top *n* features for clustering.

1 $$ {S}^2=\frac{{\displaystyle \sum }{\left(X-\overline{X}\right)}^2}{n-1} $$

**Equation 1:** Variance formula

We used NMF method for clustering, which aims to find a small number of metagenes, each defined as a positive linear combination of all the genes so that the method can approximate the mutation load of the samples as positive linear combinations of these metagenes. Mathematically, this corresponds to factoring a given non-negative matrix A of size *m × n*, into two smaller matrices, *W* ∈ ℝ^*mxk*^ and *H* ∈ ℝ^*kxn*^, with positive entries, *A* ≈ *WH* using a positive integer number *k* < *min*{*m*, *n*}. Matrix W, called as a basis matrix and has size *m × k*, with each of the k columns defining a metagene; and entry *w*_*ij*_ represents the coefficient of gene *i* in metagene *j*. Matrix H is named as coefficient matrix and has size *k × n*, with each of the m columns representing the metagene expression pattern of the corresponding sample; and entry *h*_*ij*_ represents the mutation load of metagene *i* in sample *j*. There are multiple solutions to this problem and in this study we adopt a method by Brunet et al. [22] that was shown to perform better. The solution to form factors W and H can be obtained as explained in the following. The method starts by randomly initializing the matrices W and H and iteratively updates W and H to minimize a divergence function. W and H are updated by using the coupled divergence equations shown in Equation 2.

2 $$ {W}_{ia}\leftarrow {W}_{ia}\frac{{\displaystyle {\sum}_u}{H}_{au}{A}_{iu}/{(WH)}_{iu}}{{\displaystyle {\sum}_v}{H}_{av}},\ {H}_{au}\leftarrow {H}_{au}\frac{{\displaystyle {\sum}_i}{W}_{ia}{A}_{iu}/{(WH)}_{iu}}{{\displaystyle {\sum}_k}{W}_{ka}} $$

**Equation 2:** Coupled divergence equations to update the W and H matrices

As a result of factorization, we use coefficient matrix H to group our samples into given number (*k*) of clusters. Algorithm assigns each sample according to the highest scored metagene in patients designated column in matrix H; meaning that sample *j* will be assigned to the cluster *i* if *h*_*ij*_ is the highest entry in column *j*.

To specify the optimal number of clusters (rank of clustering) and features (genes) to use in clustering, we used consensus matrix and average silhouette width of consensus matrix.

Since the NMF algorithm starts with a random initial class assignment of samples, repeated runs over the same sample set with constant input parameters may not result in the same sample assigned to the same class between the runs; however, if we observe only a little variation in these associations between runs, then we can conclude with confidence that a strong clustering was performed for this set of parameters (number of clusters and features). This idea forms the basis for our clustering performance evaluations.

Consensus matrix is a concept proposed by Brunet et al. [22] providing visual insights about the performance of clustering. The concept can be explained as follows. In each run, sample to class assignments can be represented by a connectivity matrix *C* of size *m × m* by entering *c*_*ij*_ = 1 if samples *i* and *j* are assigned to the same cluster and *c*_*ij*_ = 0 otherwise. Then the consensus matrix, $\overline{C}$, can be calculated by averaging the connectivity matrix *C* for many clustering runs. (We selected to use 100) The value in ${\overline{C}}_{ij}$ ranges from 0 to 1 and reflects the probability of samples *i* and *j* assigned to the same cluster. In the case of a stable clustering then we expect to see most of the values in $\overline{C}$ to be close to 0 or 1.

In addition to the consensus matrix, we used average silhouette width of consensus matrix (silhouette(consensus)), introduced by Rousseeuw [37], to quantitatively measure the stability of the clustering runs with different parameters. Silhouette concept is defined as follows: for each sample we can define *a* (*i*) as the average dissimilarity/distance of sample *i* with all other data within its cluster, the value of *a* (*i*) will then indicate how well the sample *i* fits into its assigned cluster by having a smaller value showing better assignment. Then we can define *b*(*i*) by the lowest average dissimilarity of sample *i* to any other cluster, that *i* is not a member. In other words *b*(*i*) indicates the average dissimilarity of sample *i* to its closest neighboring cluster or its next best fit cluster. Then the silhouette score of a sample can be calculated as in Equation 3 below. The value of *s*(*i*) can range from −1 to 1, and being close to 1 means that the sample is perfectly clustered. And average of *s*(*i*) over all the samples, named as average silhouette width, shows how well the data has been clustered.

3 $$ s(i)=\left\{\begin{array}{c}\hfill 1-\frac{a(i)}{b(i)},\  if\ \left|a\right.(i)<b(i)\hfill \\ {}\hfill \kern1em 0,\  if\left|a\right.(i)=b(i)\hfill \\ {}\hfill \frac{b(i)}{a(i)}-1,\  if\ a(i)>b(i)\hfill \\ {}\hfill \hfill \end{array}\kern0.5em \right.\ \mathrm{a}\mathrm{lso}\ \mathrm{can}\ \mathrm{be}\ \mathrm{written}\ \mathrm{a}\mathrm{s}\kern0.5em s(i)=\frac{b(i)-a(i)}{ \max \left\{a(i),b(i)\right\}} $$

**Equation 3**: Equation shows how the silhouette score of sample can be computed

We used the consensus matrix’s silhouette score to determine the optimal number of genes and clusters by iteratively running the algorithm over a range of biologically reasonable parameters (from 10 to 1000 top variant genes and from 2 to 10 clusters).

Lastly, among several implementations of NMF in various programming languages, we selected to use an R implementation of NMF, published by Gaujoux and Seoighe [38], because of its efficient and flexible parallel processing design and ease of applicability to our study.

### Characterization of clusters

To characterize the clusters we discovered, we correlated the samples in the clusters with their clinical features. We defined stage I and II as early stage and stage III and IV as late stage. The Fisher’s exact test was used to assess the stage tendency of clusters.

We compared the mutation score of genes between clusters using the Wilcoxon rank-sum test, and adjusted the multiple testing with the false discovery rate (FDR). The FDR was estimated using the Benjamini-Hochberg procedure [39]. We used the R language and environment [40] to run all the statistical tests. In addition, we performed functional analysis of the differentially mutated genes between the clusters using the Ingenuity Pathway Analysis (IPA; Ingenuity Systems Inc., Redwood, CA, USA) and the Gene Set Enrichment Analysis (GSEA) tools [23].

### Development of classification model

For running feature selection, classification model generation using ML algorithms and performance measurements, we used the Waikato Environment for Knowledge Analysis (WEKA) [41] framework, which is an open-source, Java-based framework.

For feature selection, we used the Information gain attribute evaluator [33], and Ranker algorithms implemented in Weka for evaluation and searching of the features. We used five diverse and most popular ML algorithms; namely RF [15], Naïve Bayes [31], C4.5 (named as J48 in Weka) [30], SVM [29], and KNN [32] to build classification models. For performance measurements, we used 10-fold cross-validation. In 10-fold cross-validation, patients are randomly partitioned into ten equal sized parts keeping the class ratio const1ant in each part; nine parts are used for training the classifiers and remaining part is used for testing. This procedure is repeated ten times, resulting each part is tested against the models built using other nine parts. The average of performance measurements of all ten iterations is considered as an unbiased estimate of the whole classification model. We report the performance of the classifiers using standard classification evaluation metrics, including: accuracy, sensitivity (true positive rate, TPR, also called recall), specificity (true negative rate, TNR), false positive rate, false negative rate, precision (Positive Predictive Value, PPV) and F measure (also called F1 score). In the Additional file 1: Table S1, we show (a) confusion matrix, also called contingency table, which is used to calculate performance measures, (b) values making true positives (TP), false positives (FP), true negative (TN), and false negatives (FN), and (c) the equations to calculate performance measures. In addition, we generate ROC curves, which graphically present the performance of classifiers for each class and calculate the area under the curve (AUC) as a numeric evaluation of ROC curves. Also, we would like to note that even though most of these measures initially defined for binary classification (having only two classes); they are applicable to multiclass classification by following one-verses-rest approach.

Finally, to validate the strength of the achieved prediction accuracy, we run a permutation test. For this test we generated 10,000 datasets by randomly shuffling patient labels in our dataset, while keeping the number of patients in each class constant. We run 10-fold cross-validation with RF classification algorithm together with feature selection step on these datasets, in the same way used for the real data in the study. We calculated a p-value by the number of times this validation produced a better accuracy on randomly shuffled dataset divided by 10,000.

## Abbreviations

AUC, area under the curve; BC, breast cancer; CADD, combined annotation dependent depletion; dbSNP, single nucleotide polymorphism database; EM, expectation maximization; ER, estrogen receptor; FN, false negatives; FP, false positives; GSEA, gene set enrichment analysis; HER2, human epidermal growth factor receptor 2; IHC, immunohistochemical; IPA, ingenuity pathway analysis; KNN, k-nearest neighbor; LDA, linear discriminant analysis; MAF, minor allele frequency; ML, machine learning; NCBI, National Center For Biotechnology Information; NGS, next-generation sequencing; NMF, non-negative matrix factorization; PCA, principal component analysis; PPV, positive predictive value; RF, random forest; ROC, receiver operating characteristic; SVM, support vector machine; TCGA, the cancer genome atlas; TN, true negative; TNR, true negative rate; TP, positives; TPR, true positive rate; WEKA, Waikato environment for knowledge analysis

## Additional file

Additional file 1: Supplementary results. This file contains supplementary tables and figures. Explanatory text is included in this file. (DOCX 391 kb)
