# Supplementary material for: Classification of breast cancer patients using somatic mutation profiles and machine learning approaches
Source: BMC Syst Biol. 2016 Aug 26;10(Suppl 3):62. doi: 10.1186/s12918-016-0306-z (PMC5009820; doi:10.1186/s12918-016-0306-z)
Supplement: Additional file 1: — Supplementary results. This file contains supplementary tables and figures. Explanatory text is included in this file. (DOCX 391 kb) [file 12918_2016_306_MOESM1_ESM.docx]

**Additional file 1 Tables and Figures**

| \|  \|  \| Actual label \| \| \| \| --- \| --- \| --- \| --- \| --- \| \|  \|  \| Cluster 1 \| Cluster 2 \| Cluster 3 \| \| Classified as \| Cluster 1 \| a \| b \| c \| \|  \| Cluster 2 \| d \| e \| f \| \|  \| Cluster 3 \| g \| h \| i \| | \|  \|  \|  \|  \| \| --- \| --- \| --- \| --- \| \|  \| Cluster 1 \| Cluster 2 \| Cluster 3 \| \| TP: \| a \| e \| i \| \| FP: \| b+c \| e+f \| h+i \| \| TN: \| e+f+h+i \| d+g+f+i \| d+e+g+h \| \| FN: \| d+g \| e+h \| f+i \| |
| --- | --- | --- | --- | --- | --- | --- | --- | --- | --- | --- | --- | --- | --- | --- | --- | --- | --- | --- | --- | --- | --- | --- | --- | --- | --- | --- | --- | --- | --- | --- | --- | --- | --- | --- | --- | --- | --- | --- | --- | --- | --- | --- | --- | --- | --- | --- | --- | --- | --- | --- |
| a- Confusion matrix | b- Definitions of basic measures |

| (Sensitivity) TPR: | TP/(TP+FN) |
| --- | --- |
| (Specificity)TNR: | TN/(TN+FP) |
| FPR: | FP/(FP+TN) or 1- |
| FNR: | FN/(FN+TP) |
| (Precision) PPV: | TP/(TP+FP) |
| F measure: | 2*(PPV*TPR)/(PPV+TPR) |

c- Definitions of performance measures

**Table S1:**

1. Confusion matrix showing the number of patients predicted to be in a class and actual number of patients in that class. As an example value “a” shows the number of patients correctly predicted to be in Cluster 1. And value “b” shows the number of patients that are predicted to be in Cluster 1, but they belong to Cluster 2 in reality.
2. Shows the definition of basic measures, which are used to calculate performance measures.
3. Shows the equations to be used to calculate performance measures.


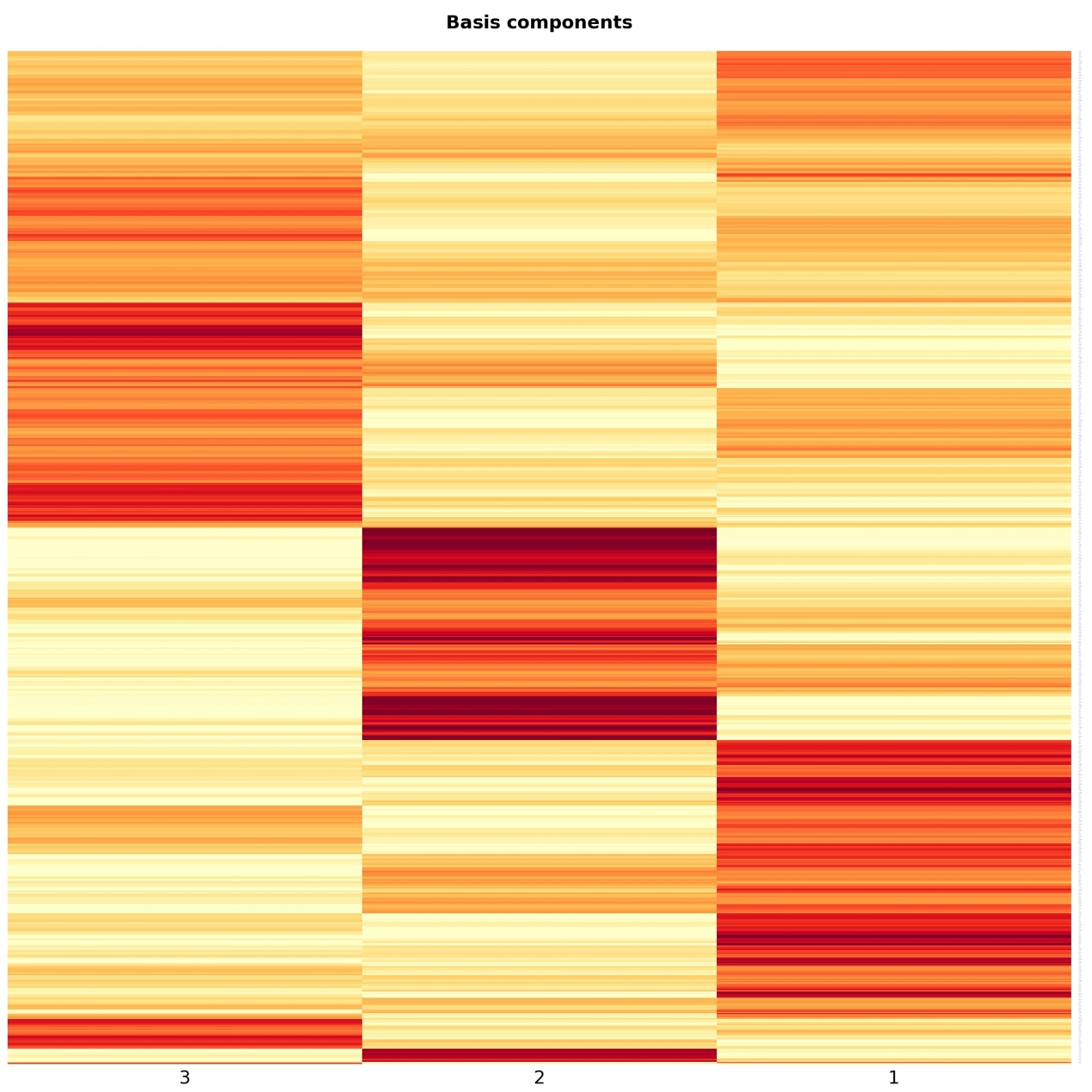


**Figure S1: Basis matrix (W)**

Basis matrix (W), which is in size of $\boldsymbol{854\times3}$, is one of the output of the non-negative matrix factorization. This matrix is not used in the scope of this work; however it could serve to the purpose of clustering genes.
